# Editorial: Pediatric Endoscopy and Sedation

**DOI:** 10.3389/fped.2022.875156

**Published:** 2022-03-15

**Authors:** Ron Shaoul, Jenifer R. Lightdale, Andrew S. Day

**Affiliations:** ^1^Pediatric Gastroenterology and Nutrition Institute, Ruth Children's Hospital of Haifa, Rambam Medical Center, Faculty of Medicine, Technion, Haifa, Israel; ^2^Division of Pediatric Gastroenterology and Nutrition, UMass Memorial Children's Medical Center and Department of Pediatrics, University of Massachusetts Medical School, Worcester, MA, United States; ^3^Department of Paediatrics, University of Otago Christchurch, Christchurch, New Zealand

**Keywords:** endoscopy, children, pediatrics, sedation, gastrointestinal

Gastrointestinal (GI) endoscopic procedures are central and critical components in diagnosing, managing and monitoring numerous pediatric conditions affecting the GI tract (1). Undergoing GI endoscopy can be uncomfortable for young patients, and typically requires sedation. Consequently, ensuring the safe and effective undertaking of procedures in children of all ages is important. In recent years, there have been numerous advances in methods and in technology that are now regular features of pediatric GI endoscopy. This Research Topic aimed to draw together a series of reports focusing on various relevant and topical aspects of endoscopy and sedation in children and adolescents.

## General Aspects of Endoscopy in Children

Cox et al. provide an excellent overview of endoscopy in children and adolescents. This review describes the history of pediatric endoscopy, as well as hints of its future advances and challenges. Within their report, the authors review aspects of sedation, common and advanced procedures, as well as complications that may occur. Practical aspects for the future of endoscopy mentioned include artificial intelligence, robot assistance, and disposable endoscopes.

Fachler et al. reviewed the yield and appropriateness of 329 endoscopic procedures in children at an Israeli children's hospital. Overall, there were no significant complications arising in this cohort. The primary indication in 88 (26%) of the children was pain: 36% of this subgroup had significant diagnostic findings. Diagnostic findings were seen in 43% of the children with other indications.

Optimal bowel preparation is critical to the performance of ileo-colonoscopy (2). While it may be the least pleasant aspect of this procedure from the patient's perspective, it is also the most important. Mamula and Nema review aspects of bowel preparation for ileo-colonoscopy in children: these include the type and method of preparation, safety, and outcomes.

The world has been rocked by the coronavirus pandemic from early 2020 to now. The ramifications of this include disruptions to regular healthcare activities, including endoscopy. Shaoul and Day provide an overview of the impacts of the pandemic upon endoscopy services. One key aspect has been the variations and constantly changing landscape over the duration of the pandemic. A number of national and international guidelines have arisen during this time. In addition, a number of novel approaches and initiatives have been developed.

## Sedation for Endoscopy in Children

Lee et al. evaluated the use of non-anesthetist administered propofol (NAAP) in 496 children undergoing endoscopic procedures at one North American hospital and compared outcomes to 433 children having their procedure under general anesthetic (GA). The adverse event rate was lower in the NAAP group, with respiratory events being particularly prominent in the GA group. The authors concluded that NAAP had an acceptable safety profile, that was similar to that seen in adults undergoing NAAP procedures (3).

A similar adverse event rate (3.8%) was observed in a second report focusing on anesthesiologist sedation regimens for endoscopy in children (Hartjes et al.). This study retrospectively evaluated outcomes in 258 children who underwent upper and/or lower endoscopy procedures. The authors highlighted wide unwarranted variations in endoscopic sedation as administered by anesthesiologists (with 29 different regimens noted), as a factor for future improvement initiatives.

Another approach to sedation for endoscopy may be hypnosis. Tran et al. reported their prospective evaluation of hypnosis in 140 children. Most (82.9%) successfully underwent endoscopy under hypnosis in combination with sedation (midazolam and/or nitrous oxide, with only 11 requiring rescheduling for GA. These results provide a promising novel approach to sedation for endoscopy in children and need to be evaluated further.

## Endoscopy in Specific Esophageal Conditions in Children

The diagnosis of eosinophilic esophagitis (EE) relies on assessment of the endoscopic appearance and evaluation of mucosal biopsies (4). Nguyen et al. review the role of endoscopy in this increasingly prevalent condition and highlight new and upcoming developments.

Esophageal atresia (EA) is a significant condition presenting at birth, requiring surgical intervention early and with life-time consequences (5). One risk is the development of anastomotic stricture, which then requires dilatation or resection. Baghdadi et al. report their experience with an early endoscopic assessment of esophageal diameter in the prediction of future need for management of stricture. One hundred and twenty-one children with EA underwent endoscopy at a median of 22 days post-operatively. Smaller anastomotic diameter was strongly associated with risk of subsequent resection (Odds Ratio of 12.9) and need for dilatations. Whilst these data need further evaluation and validation, they do provide strong support for early endoscopic evaluation as part of the routine care of these children.

## Recent Advances in Endoscopy and Therapeutic Endoscopy in Children

Since it's development and uptake, endoscopic ultrasound (EUS) has now become accepted in adult gastroenterology, but been adopted more slowly in children. Piester and Liu reviewed their collective experience over approximately two and half years. The indications and outcomes of 98 EUS procedures conducted in 72 children were reviewed. Overall, EUS was performed safely for a variety of indications. The authors also provided their perspectives of the future application of EUS in children, with mention for further exciting applications and evolutions of this methodology.

Cohen and Oliva reviewed the field of capsule endoscopy (CE) in children. Aspects covered included indications of CE and issues relating to performance in children (such as capsule placement). This review provides an excellent overview of the role of CE and pan-enteric CE in children.

Endoscopy also increasingly enables a range of therapeutic applications. Schluckebier et al. provide a comprehensive review of various therapeutic endoscopic procedures. Various future advances will continue to expand this area.

## Conclusions

Together the articles in this special issue provide important and timely updates about the current status of GI endoscopy and sedation in children and adolescents. Endoscopy has come a long way in the last decades.

The included articles also highlight many aspects of the future of endoscopy in children: these topics include machine learning/AI in endoscopy, remote control endoscopy and ultrathin endoscopy, as well as advances in therapeutic endoscopy. Other aspects of endoscopy such as green endoscopy are also very relevant to pediatric endoscopists. Endoscopy remains a key component of the pediatric gastroenterology practice now and in the coming times.

## Author Contributions

All authors listed have made a substantial, direct, and intellectual contribution to the work and approved it for publication.

## Conflict of Interest

The authors declare that the research was conducted in the absence of any commercial or financial relationships that could be construed as a potential conflict of interest.

## Publisher's Note

All claims expressed in this article are solely those of the authors and do not necessarily represent those of their affiliated organizations, or those of the publisher, the editors and the reviewers. Any product that may be evaluated in this article, or claim that may be made by its manufacturer, is not guaranteed or endorsed by the publisher.
